# Refractory Sjögren's syndrome myelopathy successfully treated with subcutaneous tocilizumab: A case report

**DOI:** 10.1097/MD.0000000000016285

**Published:** 2019-07-05

**Authors:** Yuichi Ishikawa, Koto Hattori, Junichi Ishikawa, Michio Fujiwara, Yasuhiko Kita

**Affiliations:** Department of Rheumatology, Yokohama Rosai Hospital, 3211 Kozukue-cho, Kohoku-ku, Yokohama, Kanagawa, Japan.

**Keywords:** serum amyloid A protein, Sjögren's syndrome, Sjögren's syndrome myelopathy, tocilizumab

## Abstract

**Rationale:**

It is known that 5% to 34% of Sjögren's syndrome (SS) cases are complicated by neuropathy in the form of myelitis. Although SS myelopathy (SSM) is often treated with glucocorticoid (GC) and immunosuppressants such as cyclophosphamide (CY), a therapeutic strategy for SSM has not been established.

**Patient concerns:**

A 65-year-old female was admitted with weakness and thermal hypoalgesia in the lower limbs. Four months before this admission, she showed weakness in her lower limbs and thermal hypoalgesia of bilateral upper and lower limbs. Magnetic resonance imaging (MRI) revealed that the cause of her neurological symptoms was cervical myelitis. She was diagnosed with SS because she tested positive for the ophthalmic test (Schirmer's test and fluorescent test) and for the anti-SS-A antibodies. Therefore, myelitis was thought to be a complication of SS. She was treated with GC and CY. Both neurological symptoms and MRI findings temporarily improved, and the GC dose was gradually decreased. One month before this admission, her neurological symptoms and MRI findings were exacerbated. Upon relapse of SSM, serum amyloid A protein (SAA) level was markedly elevated.

**Diagnoses:**

Based on MRI findings, the diagnosis was SSM relapse.

**Interventions:**

Treatment by subcutaneous tocilizumab (TCZ) 162 mg every two weeks was introduced.

**Outcomes:**

After introducing TCZ, her neurological symptoms and MRI findings gradually improved. SAA levels remained low. At eight months after the introduction of TCZ, the GC dose has been decreased and so far, the myelitis has not relapsed.

**Lessons:**

This case report is the first report suggesting the effectiveness of TCZ for refractory SSM. Subcutaneous TCZ might be an effective therapeutic option for treating refractory SSM when SAA levels are elevated.

## 1 Introduction

Sjögren's syndrome (SS) is a disease that causes dryness in the eyes and mouth due to the infiltration of lymphocytes into the salivary and lacrimal glands. In addition, myelitis is a known complication associated with SS,^[1]^ with 5% to 34% of SS with neuropathy cases being complicated by myelitis. Myelitis often presents as an early symptom of SS prior to the presentation of gland symptoms.^[2–4]^ Neuromyelitis optica (NMO), also known as Devic's disease, is characterized by the inflammation and demyelination of the optic nerve (optic neuritis) and the spinal cord (myelitis).^[5]^ NMO is a known complication associated with various autoimmune diseases, including SS. Some cases of myelitis complicated with autoimmune disease are thought to be related to lesions associated with NMO.^[6]^ The pathophysiology and the clinical course of SS myelopathy (SSM) and NMO have been reported to show similarities.^[2]^ Both SSM and NMO are often treated with glucocorticoids (GCs). The effectiveness of immunosuppressants such as cyclophosphamide (CY) and plasma exchange for SSM has also been reported. However, no immunosuppressive therapy has been established for either SSM or NMO.^[7–9]^ A recent study reported that the serum and cerebrospinal fluid (CSF) levels of interleukin-6 (IL-6) were elevated in the acute exacerbating period of NMO,^[10]^ indicating that IL-6 is closely involved in the pathophysiology of NMO. The effectiveness of tocilizumab (TCZ), an IL-6 receptor inhibitor, in treating NMO complicated with SS has also been reported.^[11]^ We report the case of a patient with refractory SSM who showed resistance to a combination therapy consisting of GCs and CY but was successfully treated with subcutaneous TCZ.

## 2 Case report

**Figure 1:**
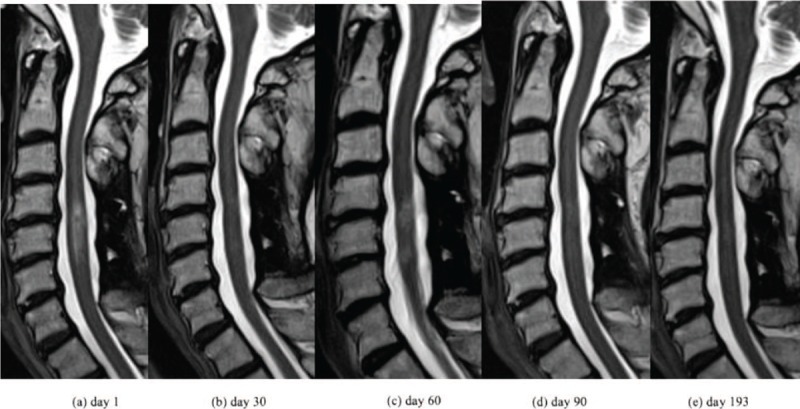
Cervical spinal magnetic resonance imaging (short-tau inversion recovery sequence). Day 1, (b) Day 30, (c) Day 60, (d) Day 90, (e) Day 193.

**Figure 2:**
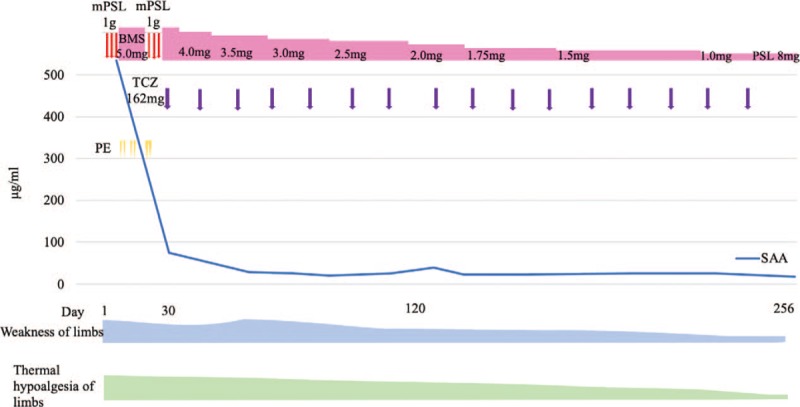
Clinical course of the patient. BMS = betamethasone, mPSL = methylprednisolone, PE = plasma exchange, PSL = prednisolone, SAA = serum amyloid A protein, TCZ = tocilizumab.

The patient was a 65-year-old Japanese female. One year before her admission to the study, she experienced dryness in her eyes and mouth. She developed weakness in her lower limbs and thermal hypoalgesia of bilateral upper and lower limbs 2 months later; these symptoms gradually worsened. Magnetic resonance imaging (MRI) revealed that the cause of her neurological symptoms was cervical myelitis. She was diagnosed with Sjögren's syndrome because she tested positive for the ophthalmic test (Schirmer's test and fluorescent test) and for the anti-SS-A and SS-B antibodies. The patient's myelitis was also thought to be related to SS. Optic neuritis, symptomatic cerebral syndrome, and brainstem syndrome, which were the diagnostic criteria for NMO, were not observed. Remission induction therapy consisting of two courses of GC pulse therapy (methylprednisolone [mPSL] at 1 g/day) and monthly intravenous cyclophosphamide (IVCY) was introduced 4 months before her admission to this study. Both neurological symptoms and MRI findings improved, and the prednisolone (PSL) dose was gradually decreased. One month before the study, the patient's muscle weakness and thermal hypoalgesia of limbs exacerbated. Further, MRI revealed a recurrence of cervical myelitis (Fig. 1a). The PSL dose was 17.5 mg/day, while 3 courses of the monthly IVCY had been administered at this time. Since it was a case of refractory myelitis that showed treatment resistance to the combination therapy of high-dose GCs and IVCY, we decided to re-introduce 2 courses of GC pulse therapy (mPSL, 1 g/day) and added 6 courses of plasma exchange (Fig. 2). However, it was expected that it would be difficult for GCs monotherapy alone to maintain remission. Therefore, a combination therapy containing an immunosuppressive agent other than IVCY was deemed more desirable. The serum amyloid A protein (SAA) levels which did not increase at the onset (SAA levels at onset were 2.6 μg/l) were elevated with the recurrence of cervical myelitis (532.4 μg/l). The CSF levels of IL-6 did not increase beyond 3.0 pg/ml since onset. The major laboratory findings at admission are described in Table 1. SAA production is induced in the liver upon stimulation by pro-inflammatory cytokines such as interleukin-6 (IL-6).^[12]^ Given the results from previous reports highlighting the involvement of IL-6 in the deterioration of the pathophysiology, we introduced tocilizumab (TCZ), an IL-6 receptor inhibitor. TCZ was administered after obtaining approval for use from the ethics committee of our hospital. Low SAA levels were maintained after introducing subcutaneous administration of TCZ (162 mg every two weeks) (Fig. 2). Although the neurological symptoms and MRI findings deteriorated temporarily (Fig. 1b and c), they gradually improved with treatment. At eight months after the introduction of TCZ, the PSL dose has been decreased to 8.5 mg/day and the myelitis has not relapsed (Fig. 1d and e). The Barthel index (total score) improved from 55 points at admission (feeding: 5, transfers from bed to chair and back: 10, grooming: 0, toilet use: 5, bathing: 0, mobility on level surfaces: 10, stairs: 0, dressing: 5, bowels: 10, bladder: 10) to 90 points after eight months of TCZ treatment (feeding: 10, transfers from bed to chair and back: 15, grooming: 5, toilet use: 10, bathing: 5, mobility on level surfaces: 15, stairs: 0, dressing: 10, bowels: 10, bladder: 10). We have not observed any serious adverse events including infections during the treatment with TCZ. Gradually, PSL administration will be decreased while that of TCZ will be continued.

**Table 1:**
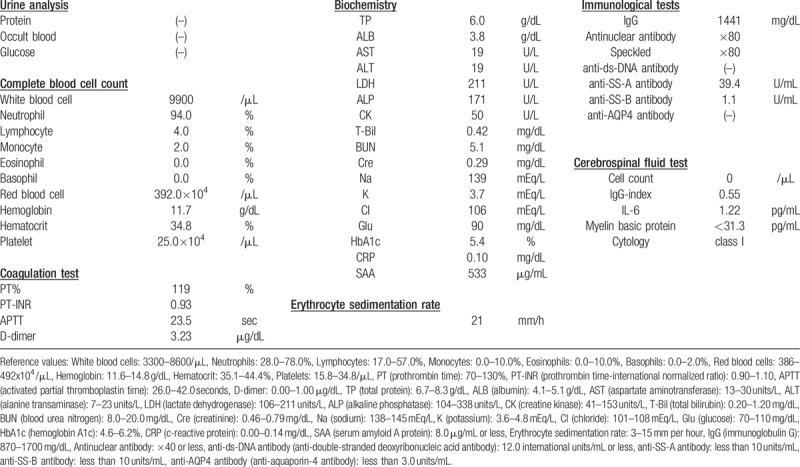
Laboratory data at admission.

## 3 Discussion

We report an efficient and satisfactory medium-term outcome in a patient with refractory SSM who was treated with subcutaneous TCZ every 2 weeks. Through continuous IL-6 inhibition therapy using TCZ, the SAA levels decreased, disease symptoms of SSM improved, and the dose of GCs could be lowered without the risk of serious adverse events such as infections. Our results suggest that IL-6 is closely involved in the etiology of SSM. It has been reported that serum and cerebrospinal fluid (CSF) levels of IL-6 are elevated in the acute period of NMO.^[10,11]^ Further, Chihara et al have reported that a B cell subpopulation exhibiting the CD19^int^/CD27^high^/CD38^high^/CD180^−^ phenotype is selectively increased in the peripheral blood of patients with NMO.^[13]^ These B cells displayed the morphological and phenotypical characteristics of plasmablasts and were further expanded during NMO relapse. The survival of plasmablasts was enhanced by IL-6, suggesting that the IL-6-dependent B-cell subpopulation is involved in the pathogenesis of NMO. This provides us with a therapeutic strategy through targeting IL-6 signaling. We expected IL-6 inhibition therapies to be effective in SSM as well, given the similarities in the etiologies of NMO and SSM.^[6]^ SAA proteins are small (104 amino acids) and have a striking relationship with the acute phase response, with serum levels rising as much as 1000-fold in 24 hours.^[14]^ SAA is not merely an acute phase protein, but activates immunocomponent cells such as monocytes, neutrophils, and lymphocytes, and induces inflammatory molecules including pro-inflammatory cytokines and matrix metalloproteinases. The production of SAA is induced by IL-6.^[15,16]^ SAA production induced in local inflamed sites contributes to the inflammatory pathology by activating immunocomponent cells.^[14]^ Although we did not observe elevated IL-6 levels in the serum or CSF, we found a marked elevation in SAA levels associated with SSM relapse. Furthermore, IL-6 inhibition therapy using TCZ was effective. Thus, IL-6 inhibition therapies might be effective when the activation of IL-6 signals is suspected (i.e., serum levels of SAA or C-reactive protein are elevated). Although TCZ has been used for treatment of NMO spectrum disease complicated with SS,^[11]^ the efficacy or safety related to the use of TCZ in treating SSM has not been reported. We considered the administration of other biological immunosuppressants such as rituximab (RTX) or tumor necrosis factor inhibitors (TNF-I). We did not choose both types of biologics because we had concerns about the safety of using RTX in this case, and about the efficacy of TNF-I in treating SS.^[17]^ As large amounts of GCs and IVCY were administered in a relatively short term, this case was considered to be associated with a high risk of infections. Therefore, it was desirable to use an immunosuppressant that promptly tapers off in concentration in the body when discontinued, in the event that a severe infection complicates the treatment. As TCZ has a short half-life (average half-life for 162 mg TCZ: 1.6 ± 0.2 days), we found TCZ to be a more suitable choice than other immunosuppressants with longer lasting immunosuppressive effects such as RTX. Moreover, RTX administration is associated with the risk of progressive multifocal leukoencephalopathy (PML).^[18,19]^ There is no report of PML occurring in patients who are being treated with TCZ. Since TNF-I has been shown to be somewhat ineffective in the treatment of the extra-glandular symptoms of SS, its administration for treating extra-glandular symptoms of SS is not recommended by the Japanese clinical practice guidelines for SS.^[20]^ Because this is a single case report, further studies with larger numbers of cases are required.

Our results suggest that TCZ might be an effective therapeutic option for treating refractory SSM when the SAA levels are elevated.

## Author contributions

**Investigation:** Yuichi Ishikawa, Koto Hattori, Junichi Ishikawa

**Supervision:** Michio Fijiwara, Yasuhiko Kita.

**Writing – original draft:** Yuichi Ishikawa
